# A dynamic source routing protocol based on path reliability and link monitoring repair

**DOI:** 10.1371/journal.pone.0251548

**Published:** 2021-05-27

**Authors:** Qing Liang, Tian Lin, Feng Wu, Fan Zhang, Wei Xiong

**Affiliations:** 1 Xi’an University of Posts and Telecommunications, School of Electronic Engineering, Xi’an, Shaanxi Province, China; 2 Xi’an University of Posts and Telecommunications, School of Automation, Xi’an, Shaanxi Province, China; Nanchang University, CHINA

## Abstract

The two most essential factors for mobile self-organizing networks applicable to drones are reliability and stability. In harsh communication environments, such as mountainous regions and natural disasters, the use of satellites and terrestrial communication stations has severe time delays due to the high speed of UAVs, resulting in frequent communication interruptions with UAVs. Therefore, UAVs need to establish self-organizing networks for communication and information sharing. High-speed movement will lead to rapid changes in the network topology, resulting in established links being in an unstable connection state and even frequent routing errors, thus preventing the establishment of stable communication links. In order to improve the communication quality of UAVs under high-speed movement, we propose a dynamic source routing protocol based on path reliability and monitoring repair mechanism (DSR-PM). The model performs data transmission by filtering the best reliability path. The link state information is monitored during transmission and broken links are repaired in time to ensure the communication stability and reliability of the links and improve the data transmission efficiency. We simulated the approach in NS2 software and the simulation results show that the DSR-PM protocol effectively reduces parameters such as overhead, packet loss and delay, improves network throughput, and provides better communication performance.

## 1 Introduction

With the development of communication technology, the current technical standards related to 5G communication have been basically completed and 5G has been commercialized in some countries and regions [1,2]. Along with the gradual solidification of 5G standardization, many researchers have started to consider the future 6G communication network [3]. 5G communication technology has achieved high-speed data transmission and its ability to provide services for different devices in different application scenarios [4]. Mobile self-organizing network is a multi-hop temporary autonomous system composed of mobile nodes with wireless transmission capability [5–7], and its application under 5G communication system is bound to be the future development direction. Routing technology, as a key technology in mobile self-organizing networks, needs to be further studied and improved to make it applicable to the communication scenario of UAVs. Due to the fast speed of UAVs, their communication status is easily affected by the complex geographical environment, and the communication through satellite or ground base station sometimes has high delay and instability. Therefore, the communication stability and reliability of routing protocols need to be further improved [8]. In order to apply UAVs to various complex communication scenarios, many researchers have conducted in-depth research and optimization of routing protocols in various scenarios.

To improve the security of communication, R. Thillaikarasi proposed an effective DSR protocol for detecting black hole attacks in wireless sensor networks [9]. This method effectively stops the black hole attack by detecting malicious nodes in the network, improves the security of communication and ensures the transmission of information. V. V. Mandhare also proposed a fast detection method based on DSR protocol to detect malicious nodes in the network [10]. Since mobile self-organizing networks are vulnerable to various attacks, malicious nodes occupy processor threads and memory space by sending multiple packets, thus preventing these nodes from receiving redundant data and information. This approach effectively identifies and detects malicious nodes, applies a reasonable node penalty system, and reconsiders node usage to ensure that each node is in the correct working state and improves the communication security of the protocol. Since wireless nodes have very limited cache space, Mandhare proposed a cache update method based on multipath DSR protocol [11]. This method is used to handle the invalid paths in the routing information to improve the cache space utilization, while recording and providing valid routing information to improve the quality of service of DSR protocol. Salari-Moghaddam also proposed a trust management system to improve the quality of service of DSR protocols [12]. This trust management approach can be used to design trust-based routing protocols to avoid attacks from malicious nodes and based on this trust management system, a method is proposed to reduce energy consumption and improve quality of service. The communication quality can be improved by increasing the packet transmission rate and residual energy while maintaining the end-to-end delay. To improve the privacy of DSR protocol, Armir Bujari proposed a security scheme for DSR protocol [13]. This scheme requires key authentication for each node in the communication, which improves the information security of the routing protocol, prevents information leakage, and ensures that the information can be delivered securely to the target node. Mythili also proposed a new secure routing mechanism, the spatial and energy-aware trusted dynamic distance DSR algorithm, for improving the survival time of wireless sensor networks [14]. Quality of service-based energy-aware routing algorithm is used to balance the effectiveness of spatial information, energy level and data quality. Based on this, a new hierarchical trust mechanism is proposed which classifies nodes based on speed, data size, energy and respective recommendations to remove nodes with abnormal behavior and increase transmission rate while ensuring security.

Because of the fast movement of UAVs, the high-speed movement inevitably leads to drastic changes in the network topology, which makes the established transmission links break and further affects the transmission performance of the protocol. The complex and variable communication environment in which UAVs are located also affects the communication performance of routing protocols, so in order to improve the communication stability and reliability of UAVs in complex environments, this paper conducts further research and optimization for this problem.

Our main contributions in this paper are as follows. First, we propose a reliability model to filter the valid paths to the target node. By computing the reliability weights of each path, we select the path with the highest reliability weights for data transmission. Secondly, in order to improve the stability of the link and the effectiveness of data transmission, we adopt a monitoring and repair mechanism to monitor the state information of the link during the data transmission process and repair the broken link in time through its neighbor nodes to provide assurance for the data transmission. Finally, the experimental results show a good improvement in the communication performance.

The rest of this article is arranged as follows: in section 2, introduced the main contents of the DSR routing protocol. In section 3, shows the optimization methods of other researchers for DSR protocol. In section 4, presents the proposed improvement methods and optimization mechanisms. In section 5, describes the simulation environment of the experiment and the analysis of the experimental results. Finally, summarize the experimental results and the methods used.

## 2 Background information

The dynamic source routing (DSR) protocol is a multi-jump on-demand routing protocol designed specifically for mobile nodes. When using the protocol, the network is fully self-organized and autonomic connected, and not use the ground base station and the satellite to build the communication between the nodes [15–17]. The source dynamic routing protocol consists of two main mechanisms, Routing discovery mechanism and routing maintenance mechanism [18].

### 2.1 Routing discovery mechanism

When a source node *s* produces a new data packet that needs to be transmitted to a destination node *d*, the source node *s* will add to a source route in the header of the data group, so that the source routing data is transmitted to the node of the destination [19]. Normally, the source node *s* can search for its local routing memory to find a route to the destination node. If no available routing is found, the source node *s* will initialize the routing to find an agreement to dynamically find a route to the destination node *d*. Therefore, the source node *s* and the destination node *d* are called initiating nodes and target nodes [20].

After the intermediate node receives the routing request, it is divided by the source node and the target node’s id and its information in the request packet, which can divide into the following situations:

1. If this node is the target node for this route, the node will send a "route response", which is collected by the reply bag from the response bag, which contains the resulting routing record, and the initiator node receives the message from the response package, and stores the information in the packet in its own routing memory for subsequent communication.
2. If the address information of this node is found in the request packet in the routing request package, the node will conclude that the request package has been received and discarded.
3. If there is no address information of this node in the routing request package, the node will record its information in the request package and then forward the routing request according to the local broadcast method until it reaches the target node.

### 2.2 Routing maintenance mechanism

When communicating, each intermediate node that sends a data packet is responsible for verifying whether the data packet can pass through the node to reach the next hop node [21,22].

**Fig 1:**
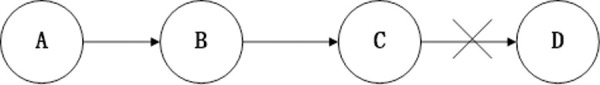
Routing maintenance example: Node C cannot forward the packet of node A to the next hop of the route.

Fig 1 shows the path from the source node A to the destination node D. Node A is responsible for verifying the link from A to B, node B confirms the link from B to C, and node C confirms the link from C to D. As can be seen from the figure above, the link D from node C to node is damaged, and the data packet cannot be passed to D through C.

The verification method can determine whether a link can carry out data transmission. In wireless networks, it is often possible to provide validation at no cost [23]. If a retransmission of a confirmation request has reached the maximum allowable number of retransmissions and no response is received, the sending node considers that the link to the next hop has been broken, removes the link from its routing memory, and returns to the source node with a routing error [24].

## 3 Related work

To reflect the contribution and value of the content of the work in this paper, we have carefully compared and analyzed it with other similar optimization methods and experimentally compared it with some of them.

In the literature [25], an MM-DSR routing protocol is proposed that collaborates with cross-layer algorithms to provide quality of service for multiple video sources on the network. The method defines two new parameters: the "Reliability Metric (RM)" and the "Movement Metric (MM)". By evaluating each path and selecting the best transmission path. However, this method is based only on the quality of service of the video source and does not guarantee its communication performance at high speed movements. In the literature [26], Vivek Sharma proposed the use of ANFIS (fuzzy inference system) to improve the DSR routing protocol for MANETs. The parameters of ANFIS system are calculated based on energy, hop count and delay to select the best routing information to ensure that the link can communicate. The paths filtered by this method, although optimal, do not take into account the speed of node movement. The network topology changes drastically when nodes move at high speeds, and more stable and reliable paths need to be selected for data transmission. In the literature [27], Hua yang proposed a DSR protocol based on continuous Hopfield neural network (CHNN-DSR) to improve the communication performance under high-speed movement of nodes. The method uses neural networks to optimize routing protocols as described in this paper to accommodate the high-speed movement of nodes, but its path selection is not the most reliable and its transmission links are susceptible to the high-speed movement of nodes, which leads to low communication quality. The literature [28] proposes a multi-intelligence based adaptive DSR (MA-DSR) protocol that optimizes the entire system through simple node-level management behaviors and incorporates factors such as signal strength into routing metrics in order to predict link disruptions before they occur. Based on this, the communication performance of the DSR protocol can be improved by updating the selection of the optimal path based on congestion measurements and energy levels of each node. The literature [29] proposes a priority-aware dynamic source routing protocol (PA-DSR), which implements a priority-aware mechanism by assigning priorities based on data rates and whose five different priorities correspond to five different connections, providing better communication compared to DSR protocols. Based on this, the communication performance of the DSR protocol can be improved by updating the selection of the optimal path based on congestion measurements and energy levels of each node. Both MA-DSR protocol and PA-DSR protocol are optimized in different aspects based on DSR protocol, but both do not consider the communication stability and reliability of the protocol when the nodes move at high speed, so the optimization method used in this paper aims to improve the communication reliability and stability when the nodes move at high speed, which has some advantages in highly dynamic networks.

We use reliability model and monitoring and repair mechanism to optimize the DSR protocol, aiming to improve the communication stability and reliability of the protocol under high-speed topology changes and provide some guarantee for UAV communication in complex environments.

## 4 Proposed work

In DSR protocol, the repair mode used by the original routing protocols is network-wide monitoring when a link is broken, which consumes a lot of energy and records a large number of useless route records, resulting in wasted buffers. The original routing protocol uses the shortest path, however, its reliability and stability cannot be guaranteed when the network topology changes rapidly. Therefore, the following improvements have been made to the DSR protocol.

### 4.1 Path reliability model

We assume the model of UAV Ad hoc network is *G* = (*V*, *E*), where *V* = {*v*_1_,*v*_2_,…,*v*_n_} is the set of nodes with valid routing information, and *E* = {*e*_1_,*e*_2_,…,*e*_n_} is the path to the destination node. The transmission delay of the *k* path between the source node *s* and the destination node *d* is defined as the length of the path, whose size is half of the total delay recorded in the routing reply packet received by the source node, denoted as

$$l(e_{k})=\frac{1}{2}D(s,d)$$ (1)

where *D*(*s*,*d*) is the cumulative transmission time required for the round-trip transmission from the source node to the destination node as recorded in the route forwarder.

Suppose the shortest delay from source node *s* to destination node *d* is called the shortest length, denoted as *l*_min(*s*,*d*)_;suppose the link from node *i* to node *j* is represented by (*i*, *j*);if *l*_min(*j*,*d*)_ ≤ *l*_min(*i*,*d*)_, this link is called the effective link. The shortest length to the destination node through link (*i*, *j*) is called the effective link length, denoted as

$$al_{\left(i,j\right)}=l_{\left(i,j\right)}+l_{\min(j,d)}$$ (2)

Suppose ${\bar{l}}_{i}$ is the average length of all valid paths connected to node *i*, then the weight of effective link (*i*, *j*) is

$$W_{e(i)}=\exp\left[\frac{-k\cdot al_{\left(i,j\right)}}{{\bar{l}}_{i}}\right]$$ (3)

Where *k* is the correlation parameter, and the value is determined according to the number of downstream links connected to the node. Then, the weight of node *i* is the sum of the weights of the effective links connected to *i* node as

$$W_{n(i)}=\sum_{j}W_{e(i,j)}$$ (4)

For any path *e*_*k*_, its path reliability is the product of the weights of all its links and all its nodes.

$$W_{e_{k}}=\prod_{i=1}^{n}W_{e(i)}\cdot \prod_{j=s}^{d}W_{n(j)}$$ (5)

According to the formula, the greater the path weight, the more reliable the path will be.

### 4.2 Monitoring and repair mechanism

After the source node receives a route reply packet from the target node, it will select the appropriate path for data communication after retrieving and copying the information in the packet. While forwarding the packet, the neighbor nodes around the path monitor the path’s status information. Once a link of the path is broken, the node at the break point will look for whether a nearby neighboring node has a path to the destination node. If a path to the destination node exists, the node upstream of the link break is immediately notified and data is transmitted via the found path. If no valid route information to the destination node is found after the neighbor node, the source node is immediately notified to reselect the route to be transmitted or to restart the route search process.

The routing monitoring repair mechanism is divided into the following steps, which will be explained in detail through examples and flow charts.

1. At the time of data transmission, start the routing monitoring repair mechanism to monitor link status information.
2. If the link is detected to be disconnected, immediately look up the routing information of the surrounding nodes. If no link break is detected, the link is in a normal transmission state.
3. If an effective route is found, data transmission will continue along the route found immediately. Otherwise, the source node is notified to reselect the path or restart the route discovery process.

**Fig 2:**
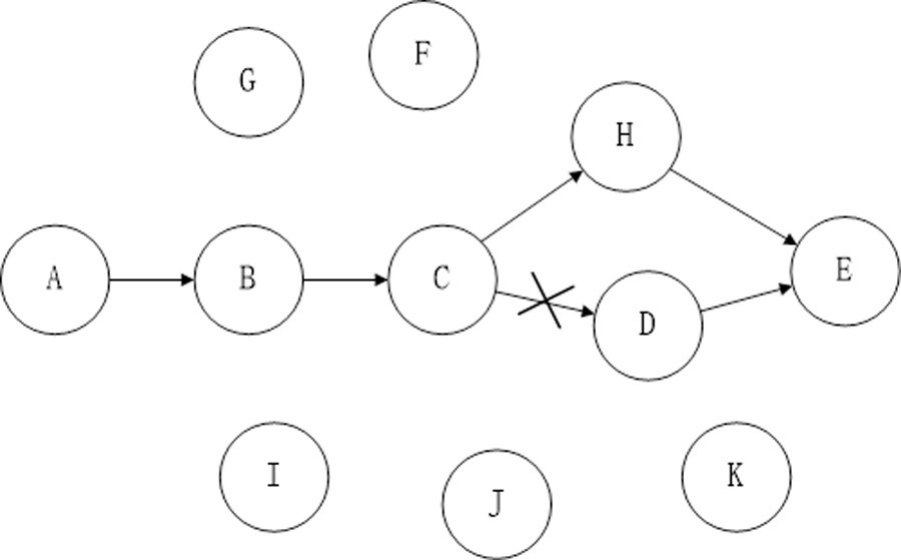
Example diagram of routing detection and repair.

We illustrate the route monitoring repair mechanism with an example. Fig 2 shows the transmission path from node *A* to *E*. When transmitting from *C* to *D*, since the *C* → *D* link is broken, the neighboring nodes detect this situation and immediately look for a valid path to the destination node *E* in the local cache. After the search, the path to the destination node is found at node *H*.Node *H* immediately notifies node *C* to continue the packet forwarding until the packet reaches the destination node *E*.

**Fig 3:**
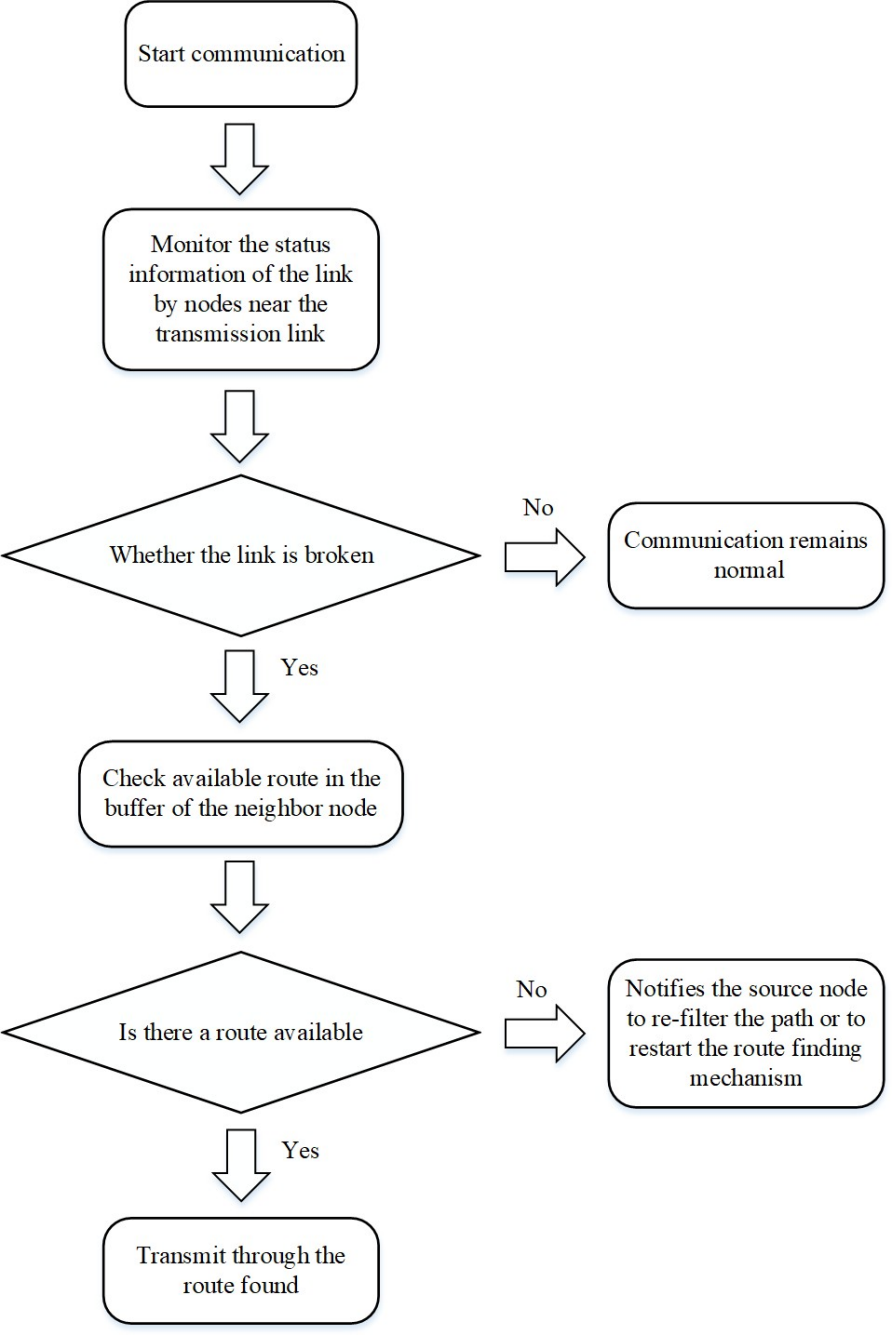
Flow chart of the monitoring repair mechanism.

In order to explain the specific steps of this mechanism more vividly, we draw the process for further explanation. Fig 3 shows the specific steps for the repair strategy.

We summarize the reliability model and the specific operational steps of the link monitoring and repair mechanism, which are listed in Table 1. The table contains the specific calculation order and routing maintenance methods.

**Table 1:** Summary of overall algorithm.

Algorithm 1 summarizes the detailed steps of the proposed model and mechanism
1. Search for routing information to the destination node 2. Use the following steps to filter effective paths 3. Calculate the transmission delay for all paths *l*(*e*_*k*_) 4. Calculate the average transmission delay for all paths $\bar{l_{i}}$ 5. Calculate the weight of each node for all paths *w*_*n*(*i*)_ 6. Calculate the weights of all paths $w_{e_{k}}$ 7. Select the path with the maximum weight value path to transmit information 8. During data transmission, use the link monitoring and repair mechanism to detect the path information 9. If the path is broken, immediately repair the link through neighboring nodes If find valid routing information and immediately transmit through the path found Else notify the source node to reselect the path or restart the routing search mechanism Else normal data transmission 10. Data transmission completed

## 5 Experimental and simulation analysis

### 5.1 The simulation environment

In order to evaluate the communication performance of the protocol in complex environments, we designed a relatively large simulation area to meet the needs of UAV scenarios in various random environments. We set the number of simulation nodes to 50, and the communication range of each node is 250 meters, which is just enough to cover the simulation area and meet the communication needs of the nodes. On this basis, we set the speed of the nodes to be 10-100m/s, which is close to the flight speed of the actual UAV. Other specific simulation parameters are shown in Table 2.

**Table 2:** Simulation environment parameters table.

parameter	numerical value
The size of scene/m	2000 × 2000
Communication radius/m	250
Packet types	CBR
Packet size/Byte	512
Number of data streams	10
Type of antenna	OmniAntenna
Wireless propagation model	TwoRayGround
Channel type	WirelessChannel
Number of nodes	50
Node maximum velocity/(m/s)	10–100
simulation time/s	100

### 5.2 Simulation analysis

#### 5.2.1 Packet loss rate

During the movement of each node, packet loss condition occurs due to network topology changes or environmental factors. Packet loss ratio is the ratio of packets lost by a node to packets sent by a node.

**Fig 4:**
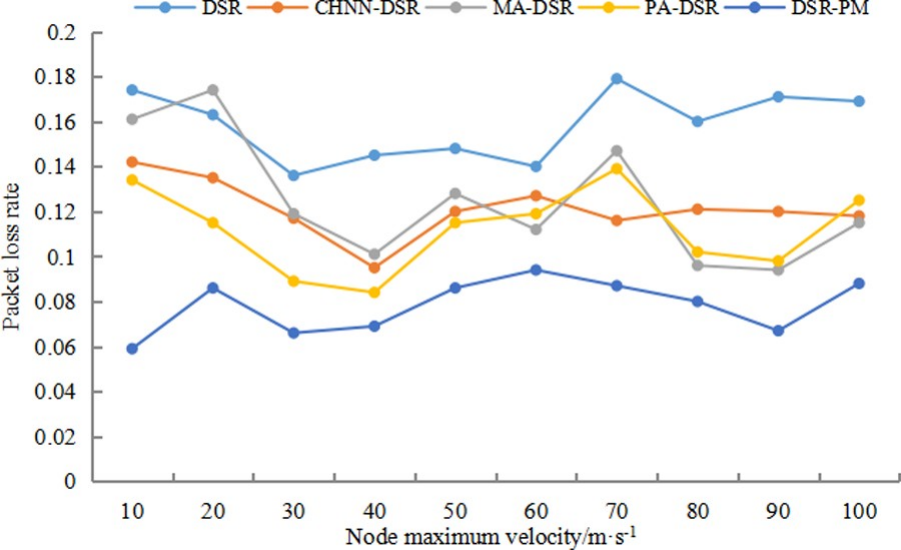
Packet loss rate comparison diagram.

Fig 4 shows the packet loss rate comparison of DSR, CHNN-DSR, MA-DSR, PA-DSR and DSR-PM protocols at speeds of 10-100m/s. Compared with the DSR protocol, the packet loss rates of all other protocols are reduced, with the DSR-PM protocol having the lowest packet loss rate. As can be seen from the figure, the packet loss rate of DSR protocol and other protocols exceeds 10% on average, however, the packet loss rate of DSR-PM protocol is less than 10%, and there is a lower number of packet loss for the same number of packets transmitted. Before performing data transmission, the reliability weight of each transmission path is calculated and the path with the highest reliability is selected for data transmission, which enhances the stability of the link, reduces the number of link breaks, and allows more valid data to be sent and improves the data transmission efficiency. During data transmission, we employ a link detection mechanism to repair broken links and resume communication in a timely manner, providing a guarantee for effective data transmission.

#### 5.2.2 Network overhead

During network transmission, some redundant data are inevitably added to the signal and the data format needs to be changed, which are necessary for transmission, and the proportion of these redundant data in the data source is called overhead.

**Fig 5:**
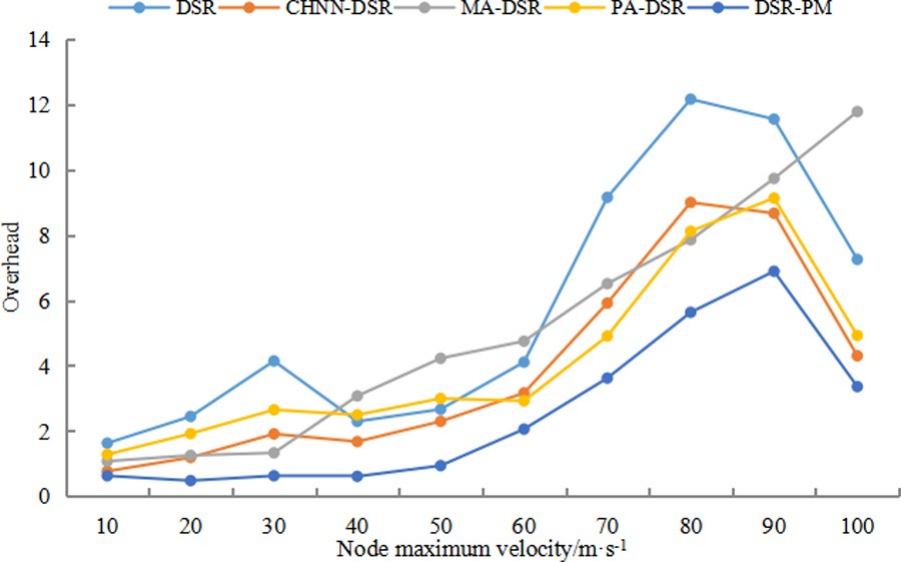
Overhead comparison diagram.

Fig 5 shows a comparison graph of the overhead of DSR, CHNN-DSR, MA-DSR, PA-DSR and DSR-PM protocols for speeds of 10–100 m/s. The overhead curves of DSR, CHNN-DSR and RE-DSR protocols are shown in Fig 5. When the speed is lower than 60 m/s, the overhead of DSR and other protocols is relatively large, while DSR-PM protocol has a lower overhead. When the speed is greater than 60m/s, the network overhead of all protocols increases sharply as the speed increases, but the routing overhead of DSR-PM protocol increases at a slower and lower rate compared to the other protocols. The higher the speed, the more drastic the network topology changes. The optimization method we use can effectively reduce the overhead of protocols moving at high speed, reduce the waste of resources while transmitting the same amount of data, and improve the transmission performance of the protocols.

#### 5.2.3 Throughput

It is necessary to evaluate the data throughput of the UAV in high-speed flight. The throughput is the total size of the data received from the starting point to the end node. To improve the high-speed communication performance of UAVs, it is important to establish a reliable and stable link to increase their throughput.

**Fig 6:**
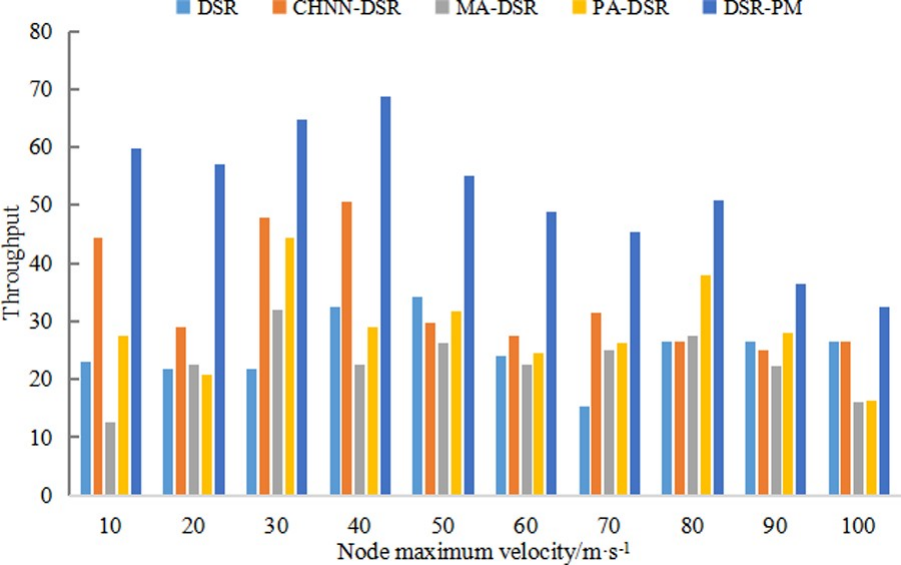
Throughput comparison diagram.

Fig 6 shows the throughput histograms of DSR, CHNN-DSR, MA-DSR, PA-DSR, and DSR-PM protocols. Both DSR and the other improved DSR protocols show significant improvements when the speed is below 50m/s, with the DSR-PM protocol taking the lead in throughput. As the speed increases, when the speed is greater than 50m/s, the network topology changes rapidly, resulting in an unstable transmission link, and its throughput gradually decreases, but the throughput of DSR-PM protocol still outperforms the other protocols. With the rapid flight of UAVs, the link state will become very unstable and the link throughput will be reduced accordingly. We adopt the link reliability model to improve the stability of the link and the corresponding link repair mechanism to extend the survival time of the link, the link can maintain a longer effective transmission time, and thus its throughput will increase accordingly.

#### 5.2.4 End-to-end delay

End-to-end delay is an important factor in mobile self-organizing networks and refers to the transmission time from the source node to the target node.

**Fig 7:**
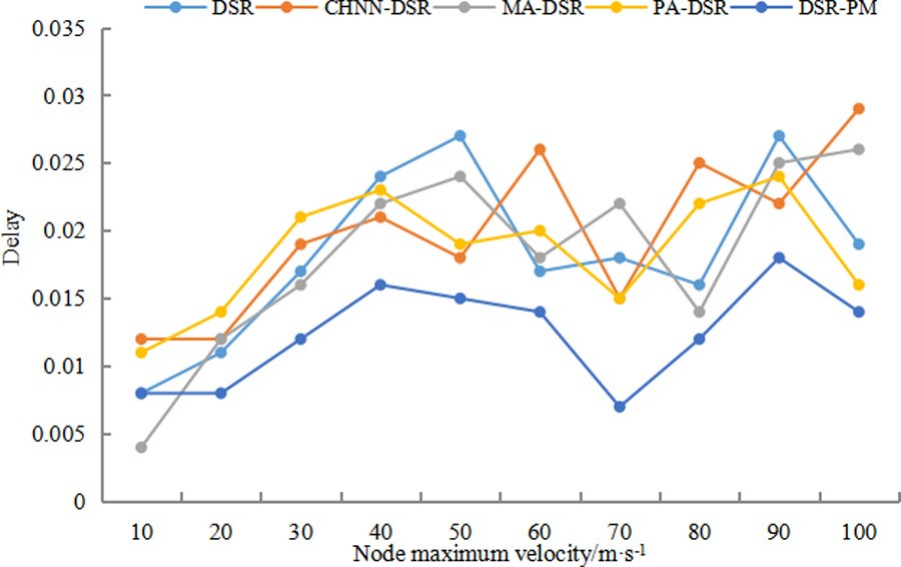
Delay comparison diagram.

Fig 7 shows the graphs of transmission delays for DSR, CHNN-DSR, MA-DSR, PA-DSR and DSR-PM protocols at speeds of 10-100m/s. It can be seen from the graph that the delay jitter is relatively sharp for all protocols, and although the DSR-PM protocol has a lower delay than the other protocols, there is still delay jitter. Nodes moving too fast can cause frequent interruptions in the established links, which leads to transmission delay jitter. Therefore, the improved method in this paper can quickly repair broken links, thus reducing the number of returns to the source node for re-path selection and reducing the transmission delay of data. Compared with returning to the source node for path rescreening, timely repair of broken links through intermediate nodes enables faster recovery of data transmission.

## 6 Conclusion and prospect

In this paper, we propose two methods to optimize the DSR routing protocol to improve the reliability and stability of this routing protocol. First, we propose a reliability model to select the most reliable route to ensure reliable communication between UAVs. Secondly, we propose a link monitoring and repair mechanism. During data transmission, broken links can be repaired by neighboring nodes in time to ensure effective data transmission. Finally, it can be concluded from the simulation experiments that the DSR-PM protocol proposed in this paper effectively improves the performance parameters such as throughput, network overhead, packet loss rate and delay of routing protocols with better transmission performance compared to DSR protocol and other improved DSR protocols.

In future work, it is very necessary to continuously optimize the protocol to ensure more stable communication performance of UAVs in high-speed motion.

## Supporting information

S1 Data Updated data file. (XLS)
